# Comparison of generalized estimating equations and quadratic inference functions using data from the National Longitudinal Survey of Children and Youth (NLSCY) database

**DOI:** 10.1186/1471-2288-8-28

**Published:** 2008-05-09

**Authors:** Adefowope Odueyungbo, Dillon Browne, Noori Akhtar-Danesh, Lehana Thabane

**Affiliations:** 1 Department of Clinical Epidemiology and Biostatistics, McMaster University, Hamilton, Canada; 2 Centre for Evaluation of Medicines, St. Joseph's Healthcare Hamilton – a Division of St. Joseph's Health System, Hamilton, Canada; 3 Department of Psychology, University of Guelph, Guelph, Canada; 4 School of Nursing, McMaster University, Hamilton, Canada

## Abstract

**Background:**

The generalized estimating equations (GEE) technique is often used in longitudinal data modeling, where investigators are interested in population-averaged effects of covariates on responses of interest. GEE involves specifying a model relating covariates to outcomes and a plausible correlation structure between responses at different time periods. While GEE parameter estimates are consistent irrespective of the true underlying correlation structure, the method has some limitations that include challenges with model selection due to lack of absolute goodness-of-fit tests to aid comparisons among several plausible models. The quadratic inference functions (QIF) method extends the capabilities of GEE, while also addressing some GEE limitations.

**Methods:**

We conducted a comparative study between GEE and QIF via an illustrative example, using data from the "National Longitudinal Survey of Children and Youth (NLSCY)" database. The NLSCY dataset consists of long-term, population based survey data collected since 1994, and is designed to evaluate the determinants of developmental outcomes in Canadian children. We modeled the relationship between hyperactivity-inattention and gender, age, family functioning, maternal depression symptoms, household income adequacy, maternal immigration status and maternal educational level using GEE and QIF. Basis for comparison include: (1) ease of model selection; (2) sensitivity of results to different working correlation matrices; and (3) efficiency of parameter estimates.

**Results:**

The sample included 795, 858 respondents (50.3% male; 12% immigrant; 6% from dysfunctional families). QIF analysis reveals that gender (male) (odds ratio [OR] = 1.73; 95% confidence interval [CI] = 1.10 to 2.71), family dysfunctional (OR = 2.84, 95% CI of 1.58 to 5.11), and maternal depression (OR = 2.49, 95% CI of 1.60 to 2.60) are significantly associated with higher odds of hyperactivity-inattention. The results remained robust under GEE modeling. Model selection was facilitated in QIF using a goodness-of-fit statistic. Overall, estimates from QIF were more efficient than those from GEE using AR (1) and Exchangeable working correlation matrices (Relative efficiency = 1.1117; 1.3082 respectively).

**Conclusion:**

QIF is useful for model selection and provides more efficient parameter estimates than GEE. QIF can help investigators obtain more reliable results when used in conjunction with GEE.

## Background

Investigators often encounter situations in which plausible statistical models for observed data require an assumption of correlation between successive measurements on the same subjects (longitudinal data) or related subjects (clustered data) enrolled in clinical studies. Statistical models that fail to account for correlation between repeated measures are likely to produce invalid inferences since parameter estimates may not be consistent and standard error estimates may be wrong [1].

Statistical methods appropriate for analyzing repeated measures include generalized estimating equations (GEE) and multi-level/mixed-linear models [2]. GEE involves specifying a marginal mean model relating the response to the covariates and a plausible correlation structure between responses at different time periods (or within each cluster). Parameter estimates thus obtained are consistent irrespective of the underlying *true *correlation structure, but may be inefficient when the correlation structure is misspecified [2]. GEE parameter estimates are also sensitive to outliers [2,3].

Summary statistics derived from the likelihood ratio test can be used to check model adequacy in cross-sectional data analyses [1,4,5]. For mixed linear models, the process is often not straightforward due to the complexities involved [6]. Model selection is difficult in GEE due to lack of an absolute goodness-of-fit test to help in choosing the "best" model among several plausible models [4,5,7]. For repeated binary responses, Barnhart and Williamson [5] and Horton et al[4] proposed ad-hoc goodness-of-fit statistics which are extensions of the Hosmer and Lemeshow method for cross-sectional logistic regression models [4,5,8].

The quadratic inference functions (QIF) – introduced by Qu et al [3] – extends the capabilities of the GEE[3]. QIF provides a direct measure of goodness-of-fit that compares the fitted model to a saturated model, gives efficient and consistent parameter estimates (irrespective of the underlying correlation structure), and yields inferences that are robust to outliers[3,9]. QIF is a relatively new methodology. A literature search in PUBMED yielded only one study that used QIF for statistical analysis [10].

The aims of this paper are: (1) to illustrate the use of QIF for longitudinal or clustered data analyses; and (2) to compare the results obtained from GEE and QIF using data from the National Longitudinal Survey of Children and Youth (NLSCY) database. In these illustrations we model the relationship between a binary response variable (parent's reports of child hyperactivity-inattention) and covariates such as child's age and gender, family functioning, maternal depression symptoms, household income adequacy, maternal immigration status and maternal educational level.

## Methods

### Overview of GEE

Marginal models are often fitted using the GEE methodology, whereby the relationship between the response and covariates is modeled separately from the correlation between repeated measurements on the same individual [2].

The correlation between successive measurements is modeled explicitly by assuming a "correlation structure" or "working correlation matrix". The assumption of a correlation structure facilitates the estimation of model parameters [2]. Examples of working correlation matrices include: exchangeable, auto-regressive of order 1 (AR(1)), unstructured, and independent correlation structures[2]. For binary data, correlation is often measured in terms of odds ratios [11]. A plausible working correlation matrix can be chosen using a visual tool known as the *lorelogram *[11].

Details of the correlation structure and response-covariate relationship are included in an expression known as the *quasi-likelihood function*[2], which is iteratively solved to obtain parameter estimates. Estimates obtained from the *quasi-likelihood function *are efficient when the true correlation matrix is closely approximated '[see Additional file 1]'. In other words, the large-sample variance of the estimator reaches a Cramer-Rao type lower bound[3] '[see Additional file 2]'.

The pros and cons of using GEE are summarized in Table 1.

**Table 1:** Summary of the pros and cons of GEE and QIF

**Attribute**	**GEE**	**QIF**
**Pros**	✔ GEE parameter estimates are efficient provided the *true *correlation structure is closely approximated. Parameter estimates are optimal in this case^2^; ✔ Modules for GEE are widely available in many statistical software applications; ✔ GEE parameter estimates are consistent irrespective of the covariance structure chosen, as long as the linear predictor and link function are **correctly **specified^1,2^	✔ Has all the pros of GEE highlighted in the adjacent column^3^; ✔ Parameter estimates are efficient irrespective of correlation structure specified^3^; ✔ Includes a "chi-squared inference function" for testing goodness-of-fit and regression misspecification. The function follows a chi-squared distribution irrespective of the specified correlation structure. P-values less than 0.5 suggests that the specified model may be inadequate to describe the observed data ^3^; ✔ The goodness-of-fit test is analogous to the LRT, thus model selection criteria such as AIC (Akaike Information Criterion) and BIC (Bayes Information Criterion) are natural extensions^3^; ✔ Gives robust parameter estimates in the presence of outliers/contaminated clusters, by using an "automatic down-weighting strategy" through a weighting matrix^3^. This property is illustrated in Qu and Song ^9^; ✔ Existence of a lower bound is guaranteed since the function has a lower bound of 0, thus solving the problem of multiple roots associated with GEE ^3^; ✔ QIF gives similar results as the GEE when the independent correlation structure is assumed^3^.

**Cons**	✔ GEE assumes that the chosen model is correctly specified. It is often difficult to assess the goodness-of-fit of models built using GEE due to lack of an inference function like the likelihood ratio test (LRT) ^7^. The likelihood function for marginal models using GEE is often difficult to evaluate and intractable, especially for data that is not normally distributed ^2^; ✔ GEE parameter estimates are sensitive to the presence of outliers as illustrated in Diggle et al ^2 ^(page 165) and Qu et al^3^; ✔ GEE parameter estimates are not efficient if the correlation structure is misspecified. Inefficient estimates may lead to faulty inferences from hypotheses tests^3^; ✔ Non-convergence of results due to lack of an objective function, and the "multiple roots" problem associated with estimating functions like the quasi-likelihood function ^29^.	✔ No software implementation available, but SAS macro available for download^28^; ✔ Is only presently applicable to three working covariance structures: Independent, Exchangeable and AR(1) ^28^;

### Overview of QIF

The QIF methodology overcomes some of the disadvantages of GEE highlighted in Table 1[3]. It is largely based on observing that the inverse of many commonly used working correlation matrices can be expressed as a linear combination of unknown constants and known matrices '[see Additional file 2]'. This linear expression is substituted back into the *quasi-likelihood function *from which an extended score vector [3] is obtained. Qu et al [3] used the generalized method of moments [12] to obtain an objective function consisting of the extended score vector and its inverse variance matrix. This function is termed the "Quadratic Inference Function", which is minimized through a numerical algorithm to obtain parameter estimates '[see Additional file 2]'.

The estimates obtained from QIF are as efficient as those from the *quasi-likelihood function *provided the *true *correlation structure is specified. Further, the estimates obtained from QIF are still efficient, even if the correlation structure is misspecified [3]. This is confirmed from simulation results obtained by Qu et al [3] comparing the simulated relative efficiency (SRE) of parameter estimators from GEE and QIF:

$$\text{SRE}=\frac{\text{mean squared error of GEE estimator}}{\text{mean squared error of QIF estimator}}.$$

Given a *true *correlation structure of AR(1) and a correlation of 0.7 between repeated observations, Qu et al [3] obtained an SRE of 1.34 (QIF more efficient) if the working correlation structure is misspecified as "equicorrelated". An SRE of 2.07 (QIF more efficient) was obtained if a true equicorrelated structure is misspecified as AR(1). SRE is in the range 0.97–0.99 if correlation structure is correctly specified, meaning GEE and QIF are similarly efficient [3]. The reliability of these simulation results is assessed in this paper.

The pros and cons of using QIF are listed in Table 1.

### The NLSCY dataset

The NLSCY dataset consists of long-term, population-based survey data collected since 1994, and is designed to evaluate the determinants of developmental outcomes of Canadian children and youth. Each two-year period from 1994 constitutes a cycle [13].

For this paper, we selected a sub-sample of children meeting the inclusion criteria outlined below.

### Inclusion criteria

Child must be four or five years old in Cycle 1 of the survey. Child must also have complete data (Cycles 1 to 4) on the following variables: hyperactivity-inattention, age, gender, family functioning, maternal (or person most knowledgeable) depression, household income adequacy, maternal immigration status and maternal educational level. The "person most knowledgeable" (PMK) is usually the child's mother [13].

### Sample size

**Figure 1:**
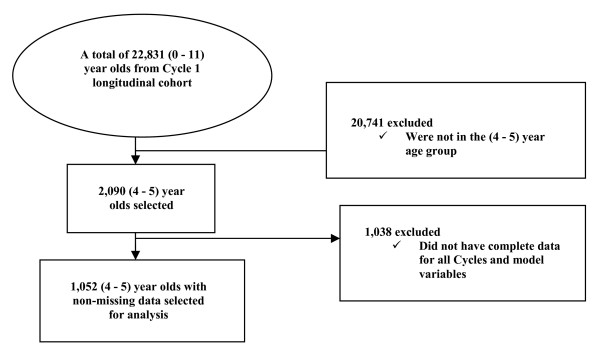
Sample selection.

From a total of 2,090 (weighted sample of 795,856) four to five year olds in Cycle 1, a sub-sample of 1,052 (weighted sample of 384,306) children met the inclusion criteria outlined above. A flowchart of this process is shown in Figure 1.

### Model variables

#### a) Response variable

The outcome of interest is hyperactivity-inattention (HI). HI is a factor measured on a 3-point Likert Scale [14] designed to assess different constructs of a child's behavior using information obtained from the PMK (or mother) [15]. The HI scale "identifies children who: cannot sit still, are restless, and easily distracted; have trouble sticking to any activity; fidget; cannot concentrate, cannot pay attention for long; are impulsive; have difficulty waiting their turn in games or groups; and cannot settle to do anything for more than a few moments" [15]. The scale is reliable with a Cronbach's alpha of 0.84 [16]. The variable – having a range of possible values between 0 and 16 – was dichotomized using specifications obtained from Offord and Lipman[17]:

$$\text{HI}=\{\begin{array}{c} 0\text{ if HI score is less than the 90th percentile i}\text{.e}\text{. child is not hyperactive-inattentive;}\\ \text{1 if HI score is higher than the 90th percentile i}\text{.e}\text{. child is hyperactive-inattentive} \end{array}$$

#### b) Independent variables

**i. Child's gender**: Male (1) or Female (0)**;**

**ii. Child's age (yr)**;

**iii. Maternal immigration status (MIS**): A parent who reported "age at immigration" was considered an immigrant (1 = immigrant, 0 = non-immigrant);

**iv. Maternal education level (ME)**: Maternal education level was categorized as (1 = those having university/college degree, 0 = those without university/college degree);

**v. Maternal depression (MD)**: Maternal symptoms of depression were measured using a shortened version of the Center for Epidemiological Depression Scale [18]. MD score ranges between 0 and 36. Scores higher than 12 were coded as (1 = moderate to severe maternal symptoms of depression), while scores 12 and below were coded as (0 = no maternal symptoms of depression). This dichotomy is consistent with previous work by To et al [19]. Cronbach's alpha value for this scale is 0.82 [13];

**vi. Family functioning (FF)**: Family functioning was measured using the 12-item general functioning sub-scale of the McMaster Family Assessment Device [20,21]. This scale measures various aspects of family functioning like problem solving, communications, roles, affective involvement, affective responsiveness and behavior control [13]. FF score ranges between 0 and 36. Families with scores greater than 14 were grouped as (1 = dysfunctional) while those with scores 14 and below were grouped as (0 = non dysfunctional), consistent with To et al [19]. Cronbach's alpha value for this scale is 0.88 [13];

**vii. Income adequacy (IA)**: Income adequacy reflects the impact of household size on family income, as defined by Statistics Canada [13]. Using a precedence from To et al [19], IA was dichotomized by combining the lowest and lower income adequacy categories to indicate (0 = low income adequacy), while the middle, upper middle and highest income adequacy groups were combined to indicate (1 = high income adequacy) [19].

#### c) Adjusted Cycle 4 longitudinal weight

The NLSCY uses a "stratified, multi-stage probability sample" survey design in which each child represents several children in the population, who are not part of the survey [13]. The longitudinal weight reflects the number of children each child represents. It is calculated as the inverse of the child's probability of selection into the survey [13]. The Cycle 4 longitudinal weights are appropriate for this analysis since these weights are adjusted for population changes between Cycle 1 and Cycle 4. We further adjusted the Cycle 4 longitudinal weight for each child in the sub-sample to reflect the approximate population of four to five year olds (i.e. adjusted total weight = 795,856). This was done to enhance the generalizability of results presented in this paper [13].

### Statistical analysis

Summary statistics are expressed as count (percent). Hyperactivity-inattention is expressed as a function of time, gender, family functioning, maternal depression, maternal immigration status, household income adequacy and maternal educational level using marginal logistic regression models in GEE and QIF (Equations 8 and 9). The "adjusted Cycle 4 longitudinal weight" is included as a weight variable in the GEE and QIF models to account for study design.

(1) Logit(μ_*ij*_) = α + β_1_*t*_*j *_+ β_2_*gender *+ β_3_*FF *+ β_4_*MD *+ β_5_*MIS *+ β_6_*ME *+ β_7_*IA*

(2) $$\text{logit(}\mu_{ij})=\alpha +\beta_{1}t_{j}+\beta_{1}^{*}t_{j}^{2}+\beta_{2}gender+\beta_{3}FF+\beta_{4}MD+\beta_{5}MIS+\beta_{6}ME+\beta_{7}IA$$

The goodness-of-fit (GOF) test in QIF is used for model assessment. We compared the fit of different models using the Q statistic [3] and its extensions such as AIC (Akaike Information Criterion) and BIC (Bayes Information Criterion). Smaller Qs, AICs and BICs indicate better fits [1,3].

QIF and GEE are compared with respect to relative efficiency of parameter estimates. We also illustrate how to use the GOF statistic from QIF in selecting an optimal working correlation matrix between AR(1) and exchangeable correlation structures. All statistical tests were conducted at 5% level of significance.

Graphs and analyses results were obtained using SAS^© ^(Version 9.1), SPSS^© ^(Version 14.0) and R (Version 2.5.1).

## Results

### Demographic characteristics (survey-weighted) and data exploration

**Figure 2:**
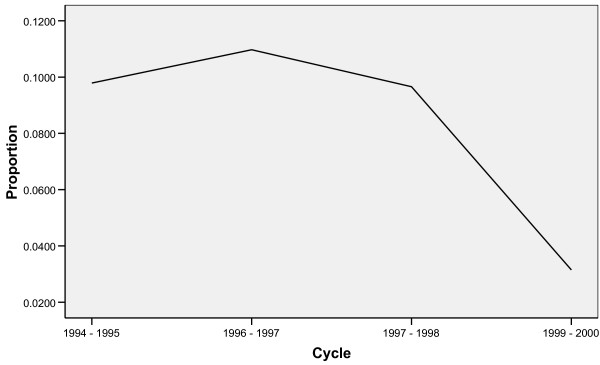
**Estimated proportion of baseline '4–5 year old' cohort with hyperactivity-inattention between 1994 and 2000**. Adjusted for "normalized" Cycle 4 longitudinal weights.

Table 2 represents the weighted frequencies of the baseline and follow-up characteristics of the study population. Figure 2 shows the estimated proportion of hyperactive-inattention among the selected cohort between 1994 and 2000. The graph is not linear. Hyperactivity-inattention appeared to diminish as children in this cohort grew older.

**Table 2:** Weighted frequencies of baseline and follow-up characteristics of the study population

**VARIABLE**	**CATEGORIES**	**CYCLE 1**	**CYCLE 2**	**CYCLE 3**	**CYCLE 4**
**Total**		795,858 (*)	795,858	795,858	795,858
**Gender**	Male	400,014 (50.3)	400,014 (50.3)	400,014 (50.3)	400,014 (50.3)
	Female	395,844 (49.7)	395,844 (49.7)	395,844 (49.7)	395,844 (49.7)
**Family functioning**	Dysfunctional	47,955 (6)	47,768 (6)	52,828 (6.6)	45,862 (5.8)
	Not dysfunctional	747,903 (94)	748,090 (94)	743,030 (93.4)	749,996 (94.2)
**Maternal depression**	Severely depressed	70,451 (8.9)	45,767 (5.8)	56,597 (7.1)	58,500 (7.4)
	Not severely depressed	725,407 (91.1)	750,091 (94.2)	739,261 (92.9)	737,358 (92.6)
**Maternal immigration status**	Immigrant	100,952 (12.7)	100,952 (12.7)	100,952 (12.7)	100,952 (12.7)
	Non immigrant	694,906 (87.3)	694,906 (87.3)	694,906 (87.3)	694,906 (87.3)
**Maternal education level**	College degree	298,839 (37.5)	330,532 (41.5)	339,078 (42.6)	301,324 (37.9)
	No college degree	497,019 (62.5)	465,326 (58.5)	456,780 (57.4)	494,534 (62.1)
**Income adequacy**	Adequate income	138,533 (17.4)	128,659 (16.2)	66,834 (8.4)	47,741 (6)
	Inadequate income	657,325 (82.6)	667,199 (83.8)	729,024 (91.6)	748,117 (94)

*Calculated based on an unweighted sample size of 1,052.

**Figure 3:**
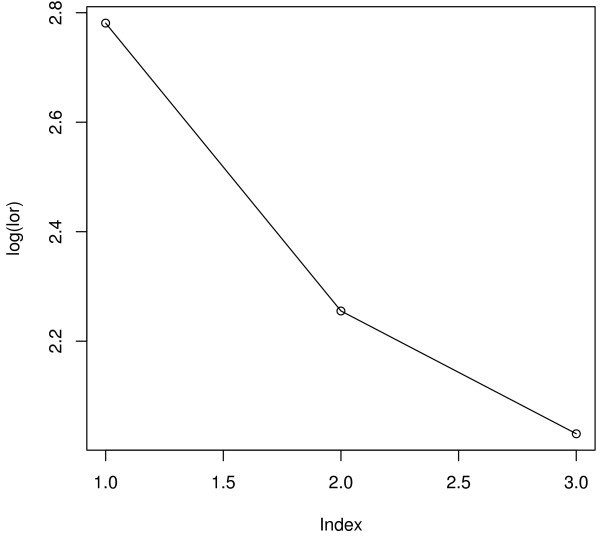
**Lorelogram of hyperactivity-inattention**. The x-axis (index) is the time-lag between two measurements. The y-axis is log odds ratio.

Figure 3 is a *lorelogram *which measures the *correlation *between repeated binary outcomes using odds ratios [11]. The x-axis (index) is the time-lag between two measurements. From Figure 3, *correlation *appears to decrease with increasing lag between repeated responses, thus an AR(1) correlation structure may be appropriate for describing the relationship between hyperactivity-inattention scores at different cycles.

### Model selection using QIF

Results in Table 3 were obtained from fitting Model (8) in GEE and QIF, and assuming an AR(1) correlation structure. GEE and QIF produce different conclusions for maternal education level and family functioning, although the odds ratios are of similar magnitudes. Figure 2 shows that a quadratic term may be required to improve model fit, but GEE does not provide a goodness-of-fit test with, for instance, the SAS^© ^implementation.

**Table 3:** Adjusted odds ratios for hyperactivity-inattention based on GEE and QIF

	**GEE**		**QIF**	
	
**Parameter**	**OR (95% CI)**	**p-value**	**OR (95% CI)**	**p-value**
**Intercept**	0.27 (0.08 to 0.96)	0.0422	0.15 (0.04 to 0.54)	0.0036
**Cycle (t)**	0.93 (0.33 to 2.63)	0.8911	0.92 (0.33 to 2.63)	0.8833
**Age (yr)**	0.90 (0.54 to 1.50)	0.6927	0.88 (0.52 to 1.47)	0.6143
**Gender (Male)**	2.08 (1.30 to 3.33)	0.0022	2.09 (1.30 to 3.36)	0.0024
**Family functioning**	2.67 (1.27 to 5.60)	**0.0097**	1.32 (0.48 to 3.63)	**0.5934**
**Maternal depression**	2.27 (1.41 to 3.66)	0.0008	3.05 (1.92 to 4.83)	< 0.0001
**Maternal immigration status**	0.51 (0.24 to 1.10)	0.0881	0.66 (0.32 to 1.40)	0.2813
**Income adequacy**	0.80 (0.46 to 1.38)	0.4249	0.95 (0.55 to 1.65)	0.8563
**Maternal education level**	0.77 (0.45 to 1.32)	**0.3476**	0.50 (0.27 to 0.93)	**0.0278**

In addition to the results obtained in Table 3, QIF – in contrast to GEE – provides direct measures of goodness-of-fit (GOF) with SAS^© ^software output to assess model adequacy [3]. QIF facilitates comparison among different plausible models using the Q statistic [3]. The Q statistic is obtained from the asymptotic limiting distribution of the quadratic inference function (QIF). Just like the likelihood ratio test, it enables one to test the null hypothesis that a simpler model is just as predictive as a saturated model. The difference between QIF (for saturated model) and QIF (for simpler model) is asymptotically chi-squared under the null hypothesis irrespective of the underlying true correlation structure. This difference is asymptotically non-central chi-squared under the local alternative hypothesis [3]. The mathematical proof and simulation results are found in Qu et al [3]. The Q statistic has properties similar to the likelihood ratio test used for generalized linear models [3]. Thus, extensions of the Q statistic such as AIC (Akaike Information Criterion) and BIC (Bayes Information Criterion) can also be used to compare the fit of different models. In comparison to a saturated model, a fitted model is considered inadequate if the p-value for the goodness-of-fit test is less than 0.05 [3].

From Table 4, GOF tests show that Model (8) is inadequate to describe the observed data (GOF statistic Q = 22.82, p = 0.0066; AIC = 40.82, BIC = 85.45). Considering the non-linearity of Figure 2, a quadratic term was added to Model (8) to obtain Model (9). Model (9) appears to provide a better fit (GOF statistic Q = 11.74; p = 0.3027; AIC = 31.74, 81.33).

**Table 4:** QIF goodness-of-fit test for model with and without quadratic term

	**With quadratic Term**	**Without Quadratic Term**
**AIC (the smaller the better)**	31.74	40.82
**BIC (the smaller the better)**	81.33	85.45
**Q (p-value)**	11.74 (0.3027)	22.82 (0.0066)

Table 5 shows parameter estimates for GEE and QIF using Model (9). The quadratic term (t^2^) is statistically significant in both GEE and QIF (p < 0.05). Also, the results from GEE and QIF appear to be in agreement in Model 9, suggesting that the results are robust. Next we provide the clinical implication of the results.

**Table 5:** Adjusted odds ratios for hyperactivity-inattention based on GEE and QIF using AR(1) (Model 9)

	**GEE**		**QIF**	
	
**Parameter**	**OR (95% CI)**	**p-value**	**OR (95% CI)**	**p-value**
**Intercept**	0.07 (0.01 to 0.33)	0.0007	0.03 (0.01 to 0.15)	<0.0001
**Cycle (t)**	4.16 (1.21 to 14.28)	0.0237	3.42 (1.00 to 11.61)	0.0486
**Cycle**^2^**(t**^2^**)**	0.73 (0.62 to 0.85)	0.0001	0.74 (0.64 to 0.86)	<0.0001
**Age (yr)**	0.91 (0.54 to 1.53)	0.7182	0.96 (0.58 to 1.59)	0.8670
**Gender (Male)**	2.08 (1.28 to 3.36)	0.0029	1.73 (1.10 to 2.71)	0.0167
**Family functioning**	2.57 (1.27 to 5.20)	0.0084	2.84 (1.58 to 5.11)	0.0005
**Maternal depression**	2.30 (1.41 to 3.74)	0.0008	2.49 (1.60 to 2.60)	0.0001
**Maternal immigration status**	0.52 (0.23 to 1.17)	0.1147	0.69 (0.35 to 1.37)	0.2937
**Income adequacy**	0.83 (0.50 to 1.38)	0.4776	0.95 (0.58 to 1.57)	0.8572
**Maternal education level**	0.74 (0.43 to 1.28)	0.2818	0.59 (0.34 to 1.02)	0.0606

### Explanation of results from Table 5 (QIF)

✔ Male children have significantly higher odds of developing hyperactivity-inattention than their female counterparts (OR = 1.73, 95% CI of 1.10 to 2.71).

✔ Children from dysfunctional families have significantly higher odds of developing hyperactivity-inattention than those from non-dysfunctional families (OR = 2.84, 95 CI of 1.58 to 5.11).

✔ Children of moderate to severely depressed mothers have significantly higher odds of developing hyperactivity-inattention than those whose mothers are not depressed (OR = 2.49, 95% CI of 1.60 to 2.60).

✔ Children of immigrants, children with mothers having university/college degree and children in the high income adequacy group have lower estimated odds of developing hyperactivity-inattention (OR = 0.69, 95% CI of 0.35 to 1.37; OR = 0.59, 95% CI of 0.34 to 1.02; and OR = 0.95, 95% CI of 0.58 to 1.57 respectively). The odds ratios in these three situations are not statistically significant.

### Choice of correlation structure in QIF

QIF facilitates an optimal choice among the available correlation structures. Assuming Model (9), Table 6 shows the results for AR(1) and exchangeable correlation structures. The results for both correlation structures are similar, but AR(1) is the more appropriate working correlation matrix from the goodness-of-fit tests in Table 7. This is also supported by the *lorelogram *in Figure 3.

**Table 6:** Adjusted odds ratios for hyperactivity-inattention using AR(1) and exchangeable working correlation structures in QIF

	**AR(1)**		**Exchangeable**	
	
**Parameter**	**OR (95% CI)**	**p-value**	**OR (95% CI)**	**p-value**
**Intercept**	0.03 (0.01 to 0.15)	<0.0001	0.02 (0.01 to 0.08)	<0.0001
**Cycle (t)**	3.42 (1.00 to 11.61)	0.0486	2.97 (0.91 to 9.67)	0.0704
**Cycle**^2^**(t**^2^**)**	0.74 (0.64 to 0.86)	<0.0001	0.71 (0.61 to 0.82)	<0.0001
**Age (yr)**	0.96 (0.58 to 1.59)	0.8670	1.13 (0.72 to 1.77)	0.6054
**Gender (Male)**	1.73 (1.10 to 2.71)	0.0167	1.83 (1.19 to 2.80)	0.0056
**Family functioning**	2.84 (1.58 to 5.11)	0.0005	2.31 (1.27 to 4.21)	0.0061
**Maternal depression**	2.49 (1.60 to 2.60)	0.0001	2.09 (1.27 to 3.46)	0.0038
**Maternal immigration status**	0.69 (0.35 to 1.37)	0.2937	0.58 (0.29 to 1.15)	0.1186
**Income adequacy**	0.95 (0.58 to 1.57)	0.8572	0.99 (0.60 to 1.61)	0.9518
**Maternal education level**	0.59 (0.34 to 1.02)	0.0606	0.68 (0.41 to 1.13)	0.1370

**Table 7:** QIF goodness-of-fit test for AR(1) and exchangeable working correlation structures

**Correlation structure**	**AR(1)**	**Exchangeable**
**Q (p-value)**	11.74 (0.3027)	12.89 (0.2298)
**AIC (the smaller the better)**	31.74	32.89
**BIC (the smaller the better)**	81.33	82.48

### GEE Versus QIF (Relative efficiency)

We compared the efficiency of parameter estimates from QIF and GEE using:

$$\begin{array}{c} \text{Relative Efficiency (RE)}=\frac{\text{mean square error of estimate from GEE }}{\text{mean square error of estimate from QIF}}\\ =\frac{\text{trace of covariance matrix of parameter estimates from GEE}}{\text{trace of covariance matrix of parameter estimates from QIF}}\\ =\frac{\text{sum of squares of SEs from GEE estimates}}{\text{sum of squares of SEs from QIF estimates}} \end{array}$$

provided the estimates are *unbiased estimates *of the parameters of interest [22]. This definition of RE generalizes to situations in which there are multiple parameters to be estimated. Thus from Table 8 with AR(1) correlation structure, one obtains

**Table 8:** Adjusted odds ratios and SEs for hyperactivity-inattention using AR(1) in GEE and QIF

	**GEE**		**QIF**	
	
**Parameter**	**OR**	**SE**	**OR**	**SE**
**Intercept**	0.07	0.7868	0.03	0.7560
**Cycle**	4.16	0.6298	3.42	0.6235
**Cycle**^2^**(t**^2^**)**	0.73	0.0791	0.74	0.0750
**Age (yr)**	0.91	0.2667	0.96	0.2574
**Gender (Male)**	2.08	0.2453	1.73	0.2287
**Family functioning**	2.57	0.3587	2.84	0.2994
**Maternal depression**	2.30	0.2485	2.49	0.2267
**Maternal immigration status**	0.52	0.4106	0.69	0.3490
**Income adequacy**	0.83	0.2777	0.95	0.2523
**Maternal education level**	0.74	0.2598	0.59	0.2819

RE = 1.1117.

Using exchangeable working correlation, RE is 1.3082 (see Table 9). This implies that QIF parameter estimates are more efficient than GEE estimates assuming AR(1) or exchangeable correlation structures. This is consistent with the simulation results obtained by Qu et al [3].

**Table 9:** Adjusted odds ratios and SEs for hyperactivity-inattention assuming exchangeable working correlation structure in GEE and QIF

	**GEE**		**QIF**	
	
**Parameter**	**OR**	**SE**	**OR**	**SE**
**Intercept**	0.06	0.8157	0.02	0.7026
**Cycle**	3.90	0.6393	2.97	0.6019
**Cycle**^2^**(t**^2^**)**	0.72	0.0794	0.71	0.0756
**Age (yr)**	0.95	0.2789	1.13	0.2304
**Gender (Male)**	2.04	0.2585	1.83	0.2177
**Family functioning**	2.35	0.3774	2.31	0.3056
**Maternal depression**	2.04	0.2788	2.09	0.2556
**Maternal immigration status**	0.54	0.4452	0.58	0.3505
**Income adequacy**	0.90	0.2607	0.99	0.2494
**Maternal education level**	0.74	0.2880	0.68	0.2563

*Calculated based on an unweighted sample size of 1,052.

## Discussion

We have illustrated some desirable features of the QIF in modeling longitudinal or clustered data. QIF provides a direct goodness-of-fit statistic that follows a chi-squared distribution irrespective of the underlying true correlation structure [3]. The goodness-of-fit statistic from QIF also facilitates an optimal selection of correlation structure among several plausible choices. It would be interesting to compare the goodness-of-fit tests provided by QIF to those provided by Barnhart and Williamson [5] and Horton et al [4] in GEE. Overall, we obtained similar parameter estimates from GEE and QIF analyses of the NLSCY data. Our results were consistent with the findings by Qu et al [3] showing the greater efficiency of parameter estimates from QIF in comparison to GEE. We could not verify the robustness of QIF to the presence of outliers due to strict ethical guidelines regarding the use of the NLSCY dataset. The risk of disclosure of sensitive data may be higher when outliers are selected for sensitivity analysis.

One of the strengths of this study is the longitudinal nature of the NLSCY dataset. However, we caution the readers in interpreting the results – dichotomizing the primary outcome hyperactivity-inattention score may result in loss of information. Understanding the factors that are predictive of hyperactivity-inattention will help stakeholders develop programs to mitigate the effects of such factors, with the aim of raising children that are healthy members of the society.

The use of "complete case analysis" in the illustrative example is a limitation of this study. QIF – like standard GEE models – requires the assumption that missing values are "missing-completely-at-random" (MCAR) for complete case analysis [30]. There are methods available for assessing this assumption or incorporating missingness into statistical models, but missing value analyses was not the aim of this project.

The QIF methodology is relatively new and not available in any statistical software as a built-in routine. The SAS macro to carry out the procedure is available for download, but users without adequate programming skills may find the process a bit difficult. Also, the QIF macro can only handle three correlation structures at the moment. More research is being done to incorporate other commonly used structures into the methodology [28].

## Conclusion

QIF is useful for model selection and provides more efficient parameter estimates than GEE. QIF can help investigators obtain more reliable results when used in conjunction with GEE. The QIF methodology may eventually become a replacement for GEE due to its desirable characteristics as highlighted in this paper.

## Competing interests

The authors declare that they have no competing interests.

## Authors' contributions

AO and LT conceived the study. LT, NA–D and DB participated in the design of the study. Data acquisition and cleaning were done by AO and DB. AO conducted data analysis and wrote initial draft of manuscript. Results of data analysis were interpreted by AO and LT. NA–D, LT and DB reviewed and revised the manuscript for important statistical and subject-matter content. All authors read and approved the final manuscript.

## Pre-publication history

The pre-publication history for this paper can be accessed here:



## Supplementary Material

Additional file 1 Glossary of terms. Provides the definitions of statistical terms used throughout the manuscript. Click here for file

Additional file 2 GEE and QIF theory. Provides a brief review of the mathematical theory behind GEE and QIF. Click here for file
