# Supplementary material for: Comparison of generalized estimating equations and quadratic inference functions using data from the National Longitudinal Survey of Children and Youth (NLSCY) database
Source: BMC Med Res Methodol. 2008 May 9;8:28. doi: 10.1186/1471-2288-8-28 (PMC2396173; doi:10.1186/1471-2288-8-28)
Supplement: Additional file 1 — Glossary of terms. Provides the definitions of statistical terms used throughout the manuscript. [file 1471-2288-8-28-S1.doc]

# APPENDIX A

## Glossary of terms:

- **Consistency:** An estimator is consistent if it converges to the “true” value as the sample size becomes infinite ^22^;
- **Efficiency:** The variance of an estimator is always greater than or equal to the Cramer-Rao lower bound ^22^. It is desirable that the variance of an estimator be small. An estimator is efficient if its variance equals the Cramer-Rao Lower Bound ^22^.
- **Relative Efficiency:** If$E_{1}$and$E_{2}$are estimators of a parameter$\theta$.The relative efficiency$E_{2}$of and$E_{1}$is defined as:

$\begin{matrix} \text{Relative Efficiency}=\frac{\text{mean square error of }E_{2}\text{ }}{\text{mean square error of }E_{1}} \\ \text{ }=\frac{E\left[\left(E_{2}-\theta\right)^{2}\right]}{E\left[\left(E_{1}-\theta\right)^{2}\right]} \end{matrix}$

$E_{2}$is more efficient than$E_{1}$if the relative efficiency is less than unity. The MSE consists of two components measuring bias and precision of estimators, e.g.

$$E\left[\left(E_{2}-\theta\right)^{2}\right]=E\left[\left(E_{2}-E(E_{2})+E(E_{1})-\theta\right)^{2}\right]=Var(E_{2})+\left(Bias_{\theta}E_{2}\right)^{2}$$
