# Supplementary material for: Comparison of generalized estimating equations and quadratic inference functions using data from the National Longitudinal Survey of Children and Youth (NLSCY) database
Source: BMC Med Res Methodol. 2008 May 9;8:28. doi: 10.1186/1471-2288-8-28 (PMC2396173; doi:10.1186/1471-2288-8-28)
Supplement: Additional file 2 — GEE and QIF theory. Provides a brief review of the mathematical theory behind GEE and QIF. [file 1471-2288-8-28-S2.doc]

# APPENDIX B

## GEE Theory

The average response of the population (or sub-population) of interest is related to the covariates through an equation of the form

$$\eta\left(\mathbf{\mu}_{i}\right)=\mathbf{X}_{i}^{T}\mathbf{\beta};$$

where$\mathbf{\mu}_{i}=E\left(\mathbf{Y}_{i}\right)$, $\mathbf{Y}_{i}$is a vector of responses for subject$i$,$\mathbf{X}_{i}$ is the matrix of covariates for subject$i$,$\mathbf{\beta}$ is a vector of parameters, and $\eta$is a link function. For instance,$\eta$is the logarithmic function for counts data, and the logit function for binary data.

The regression coefficients in$\mathbf{\beta}$are interpreted as those of cross-sectional studies ^2^.

**Working correlation matrix:**

The correlation between successive measurements is modeled explicitly by assuming a “correlation structure” or “working correlation matrix”. The assumption of a correlation structure facilitates the estimation of model parameters ^2^. Examples of working correlation matrices include: exchangeable; auto-regressive of order 1 (AR(1)); unstructured; and independent correlation structures^2^.

**Estimation:**

The parameter estimates are obtained by iteratively solving the estimating equation$S_{\mathbf{\beta}}\left(\mathbf{\beta},\mathbf{\alpha}\right)$given by

$S_{\mathbf{\beta}}\left(\mathbf{\beta},\mathbf{\alpha}\right)=\sum_{i} \left(\frac{\partial\mathbf{\mu}_{i}}{\partial\mathbf{\beta}}\right)^{T}Var\left(\mathbf{Y}_{i}\right)^{-1}\left(\mathbf{Y}_{i}-\mathbf{\mu}_{i}\right)=0$;

where$\mathbf{\alpha}$is a nuisance parameter from the working correlation matrix. Equation (2) is a multivariate version of the quasi-likelihood function ^2^.

The variance of$\mathbf{Y}_{i}$is expressed as

$Var\left(\mathbf{Y}_{i}\right)=\mathbf{G}_{i}^{\frac{1}{2}}\mathbf{R}_{i}(\alpha)\mathbf{G}_{i}^{\frac{1}{2}}\Phi$;

where$\mathbf{G}_{i}$is a diagonal matrix with elements$\mathrm{var}\left(y_{ij}\right)$,$j$is the time of measurement,$\mathbf{R}_{i}$is the working correlation matrix and$\Phi$is a constant that corrects for over-dispersion ^2^. The variance of$\mathbf{\beta}$is estimated using a robust “sandwich estimator”, and inferences are made using Wald statistics ^1, 2^.

## QIF Theory

The QIF methodology is largely based on observing that many of the commonly used working correlation structures can be written as:

$\mathbf{R}^{-1}\approx\sum_{i=0}^{k} a_{i}\mathbf{M}_{i}$;

where the $\mathbf{M}_{i}\text{'s}$are known matrices and the$a_{i}\text{'s}$are unknown constants ^3^. For instance, an exchangeable correlation matrix with 1’s on the diagonal and off diagonal elements$\alpha$may be estimated by

$$\mathbf{R}^{-1}=a_{0}\mathbf{M}_{0}+a_{1}\mathbf{M}_{1}$$

where$\mathbf{M}_{0}$is the identity matrix,$\mathbf{M}_{1}$is a matrix with diagonal elements 0 and off diagonal elements 1, $a_{0}=-\left\{\left(n-2\right)\alpha+1\right\}/k_{1}$,$a_{1}=-\alpha/k_{1}$,$k_{1}=\left(n-1\right)\alpha^{2}-\left(n-2\right)\alpha-1$and$n$is the dimension of the correlation matrix ^3^.

Equation (4) is substituted into Equation (2) to obtain

$S_{\mathbf{\beta}}\left(\mathbf{\beta}\right)=\sum_{i=1}^{N} \left(\frac{\partial\mathbf{\mu}_{i}}{\partial\mathbf{\beta}}\right)^{T}\mathbf{G}_{i}^{-\frac{1}{2}}\left(a_{0}\mathbf{M}_{0}+\ldots+a_{k}\mathbf{M}_{k}\right)\mathbf{G}_{i}^{-\frac{1}{2}}\left(\mathbf{Y}_{i}-\mathbf{\mu}_{i}\right)$.

Parameter estimation is based on an *extended score vector* $\mathbf{S}_{N}$^3^ defined as:

$\mathbf{S}_{N}(\mathbf{\beta})=\frac{1}{N}\sum_{i=1}^{N} \mathbf{S}_{i}(\mathbf{\beta})=\frac{1}{N}\left(\begin{matrix} \sum_{i=1}^{N} \left(\frac{\partial\mathbf{\mu}_{i}}{\partial\mathbf{\beta}}\right)^{T}\mathbf{G}_{i}^{-\frac{1}{2}}\mathbf{M}_{0}\mathbf{G}_{i}^{-\frac{1}{2}}\left(\mathbf{Y}_{i}-\mathbf{\mu}_{i}\right) \\ \vdots\\ \sum_{i=1}^{N} \left(\frac{\partial\mathbf{\mu}_{i}}{\partial\mathbf{\beta}}\right)^{T}\mathbf{G}_{i}^{-\frac{1}{2}}\mathbf{M}_{m}\mathbf{G}_{i}^{-\frac{1}{2}}\left(\mathbf{Y}_{i}-\mathbf{\mu}_{i}\right) \end{matrix}\right)$;

where (5) is a linear combination of the elements of (6). Using the generalized method of moments ^12^, Qu et al ^3^ defines the QIF as

$Q_{N}(\mathbf{\beta})=N\mathbf{S}_{N}^{T}\mathbf{C}_{N}^{-1}\mathbf{S}_{N}$;

where

$\mathbf{C}_{N}=N^{-1}\sum_{i=1}^{N} \mathbf{S}_{i}\left(\mathbf{\beta}\right)\mathbf{S}_{i}\left(\mathbf{\beta}\right)^{T}$.

Parameter estimates are obtained by minimizing (7) through a numerical algorithm ^3^.
